# Typho-Malarial Fever

**Published:** 1885-04-20

**Authors:** J. C. B. Renfro

**Affiliations:** LaGrange, Texas


### TYPHO-MALARIAL FEVER.

By J. C. B. RENFRO, M.D., LaGrange, Texas.

The above name is given to a fever which prevails more or less in all malarious regions. It seems to be a kind of amalgam fever, or where malarial and typhoid fever prevail at the same time in the same patient, each fever running its course. The malarial fever predominates at the outset of the attack and afterward the typhoid symptoms set in. The malarial symptoms are generally of the remittent type: paroxysms every twenty-four or forty-eight hours. The typhoid symptoms are all those connected with typhoid fever, and sometimes throughout the disease there seems to be a commingling of the two diseases in the same patient at the same time. In those cases that prove fatal, the same lesions are found as in true typhoid fever.

Those attacked with this fever usually commence with a chill, followed with high fever, temperature running as high as 103° to 106° F., when, after the usual period of from three to five hours, the sweating stage sets in, followed by the usual symptoms of simple remittent fever, general malaise, bleeding at the nose, profuse perspiration, a lowering of the temperature down to almost normal. This condition may last for several days, paroxysms occurring every twenty-four or forty-eight hours, running the same course as before, until finally the typhoid symptoms predominate and run the usual course, there being a difference of one to three degrees of the temperature morning and evening, the evening being the highest. The tongue is usually heavily furred, with tip and edges red. Sometimes the coating is slight, with the papillæ projecting so that it resembles the strawberry tongue. The bowels at times are loose; at others, constipated. The fœcal discharges are usually of a thin, dark color and offensive; more or less tympanites in the latter stage of the disease, tenderness over the bowels and gurgling about the illio coecal valve. Enlargement of the liver and spleen sometimes comes up during the course of the disease. Intense anorexia and a desire for cooling and acid drinks prevail throughout the disease. The appetite is usually very poor, with a loathing for most all kinds of food. The urine is very high colored, charged with bile, acids, and sometimes a trace of albumen in it. The cheeks always flush when the temperature begins to rise, and will last until the temperature begins to fall or sink lower, and then the flush fades away, leaving the pale, sallow hue characteristic of true typhoid fever. As to diet during the course of the disease, I usually recommend milk when it agrees with the patient; when this does not agree with them, oyster soup, or the concentrated essence of beef with soft-boiled eggs, etc. If there should be indications of asthenia or failure of the heart's action, I use alcoholic stimulants very freely, of which I prefer good, imported cognac brandy, and, if necessary, combine same with carbonate of ammonia, and it may be necessary to use digitalis to strengthen and sustain the heart's action; at all events the diet should be mild and as easily digested as possible. Patient should be careful as to diet, etc.

### PATHOLOGICAL CHARACTER AND CAUSATION.

This fever is due to poison known as malaria and the special cause of typhoid. It seems that these fevers follow in the wake of the malarial fevers, and generally come up in the latter part of the summer and during the autumnal months. I think there is an atmospheric influence that breeds and develops these fevers. I have observed this phenomena since the year 1875, that when drouth prevails and the evaporation of moisture from the earth gets beyond a certain point these fevers develop, sometimes in their worst forms, amounting almost to an epidemic and malignant in their character, and vice versa, after an extended drouth, and then floods of rain saturating the earth thoroughly, that this fever also develops, and the malarial aspect of the disease is of more grave importance, requiring more prompt and energetic treatment to subdue it.

### DIAGNOSIS.

"Typho-malarial fever is discriminated, on the one hand, from simple remittent fever by the characteristic events of typhoid fever, viz.: the abdominal symptoms, the eruption in some cases, the earlier occurrence of ataxic phenomenal epistaxis and occasional intestinal perforation. It is discriminated, on the other hand, from unmixed typhoid fever by the characteristic events of periodical fever, viz.: remissions, gastric symptoms, jaundice, and the eventuation, in some cases, in intermittent fever." Topho-malarial, after the first few days, if proper treatment be applied, will run almost the usual course of true unmixed typhoid, with this difference. however: on alternate days the temperature is almost always from one to two degrees F. higher; in true typhoid, never. In typho-malarial fever the temperature is usually higher the first few days, then sinks to a level with a gradual rise of from two to three degrees F. in the end of the second week or beginning of the third; then it usually remains about the same for about one week, then a gradual decline until convalescence is fully established. The tongue will become clean and resume its normal appearance, and convalescence ensues proper, unless a relapse should occur from some imprudence on the part of the patient. Sometimes the tongue will clean off and assume a glossy appearance; when this is the case convalescence will always be protracted. Sometimes it is almost impossible to diagnose a case of typho-malarial fever the first week of the disease.

There is one point that I have noticed more particularly in typho-malarial fever, and that is a flacidity of the veins of the back of the hand with a purplish hue of same.

In some cases the circulation and pulse are both depressed, and in others the pulse is full and bounding. Coliquitive sweats appear in some cases; in others the skin is dry and burning hot most of the time. In some cases coliquitive diarrhœa prevails; in others cathartics and laxatives must be used.

### PROGNOSIS.

In almost all cases where the complications are not too grave, the prognosis is favorable. I have noticed this, however: when there is a depression in the circulation and capillary congestion remaining for any length of time, pulse being low and the temperature running very high, and not an equal difference between the temperature and pulse, the prognosis is always grave in those cases; but under favorable circumstances, when there is no congestion of any internal organ early in the disease, no hemorrhage setting up from the bowels, and all the functions acting properly and evenly, running a regular course, the prognosis is always favorable. A sudden fall of the temperature is always indicative of hemorrhage from the bowels. Coliquitive diarrhœa and sweats are also grave symptoms, as also petechia and echymosed spots on the cutaneous surface. Loathing of food during the entire course of the disease is also of grave importance, while, on the other hand, an appetite to take on some food every day is a very favorable condition, and is indicative that the patient is not likely to die of asthenia. Upon the whole, taken as a general rule, the prognosis, with proper treatment, is favorable. Bronchitis or pneumonia, setting up as a sequel, is also a very unfavorable phenomena. I have seen a very high state of inflammation of the bowels set in the tenth day and terminate fatally in a few days afterwards.

### TREATMENT.

I now approach a point wherein we may all differ in some degree, but I hope not materially. We may differ as to therapeutics in the choice of our remedies, as to which are the best, but in the main, if we understand the disease thoroughly, we will treat it similarly. And first, as we have a mixed disease, or two diseases, to contend with in our patient, at the same time we must keep this in view; and as the malarial feature is predominant in the outset, I think the rational treatment would be, or is, to subdue all the malarial influence exerted on the economy, as far as we can, with the remedies usually used to subdue simple remittent fever, always keeping in view the condition of our patient and endeavor to irritate the bowels as little as possible. This I always do with broken doses of mercury until the secretions are thoroughly aroused, using some sedative mixture, say aconite fl. ext., one drop in water every hour until the remission occurs, then large and full doses of quinia sulph. until cinchonism is produced; then a quietus in the patient's condition is always produced, after which the typhoid symptoms will manifest themselves. I then change the treatment unless symptoms predominate demanding calomel and quinine. I seldom use it any more in the case. If pronounced intermissions should occur, we may try the quinine again, but usually it will be of little avail. It generally will produce gastric and intestinal irritation, which we should avoid as far as possible. I have myself thought quinia indicated in the latter course of the disease, and have given it, but most always with deleterious results to my patient, the next day the temperature rising higher by one or two degrees than before the administration of the drug. After the malaria is subdued in the system, but little medication is indicated usually. Watch the patient closely, and the disease from localizing itself on any of the internal organs. I usually place my patients on a compound turpentine emulsion, consisting of

℞ Oleum terebinth........................................ʒ ij

Potassa chlor........................................ʒ j

Acid hydrochloric........................................M xxx

Pul. gum acac........................................ʒ vi

Aqua pura q. s. to make a 6 oz. mixture.

Of this, give to an adult one tablespoonful every four hours. In the interim give one drop of fl. ext. aconite every hour to control the circulation. Also,

℞ Sodæ salicylate........................................ʒ vj

Aqua aromatica........................................℥ vj.

M. Sig. 1 tablespoonful every five hours.

The theory of this treatment is this: the emulsion keeps the secretions aroused, and the tongue seldom becomes dry. The salicylate of sodæ acts as an antipyretic, antiseptic and tonic, while the aconite controls the circulation. I seldom change this treatment throughout the typhoid stage; that is, generally. Of course, complications sometimes come up calling for other remedies, such as too frequent stools, coliquitive sweats and asthenia. The first I usually control with

℞ Doveri pul........................................ grs. xx
Bismuth subnit........................................grs. xl.
M. Ft. Chart No. iv.
Sig. 1 powder every four hours, as long as necessary.

The other two symptoms I usually control with alcoholic stimulants. Should hemorrhage occur from the bowels, it is usually easily controlled by fl. ext. ergot and bismuth, combined or in conjunction, a few doses of which generally answers the purpose. Of course, complications will sometimes come up calling for other remedies, of which we must be our own judge. In convalescence, tinct. of bark, preparations of iron and a nourishing diet is indicated, and should be taken in moderation.—*Trans. Tex. Asso, 1884.*

